# Supplementary material for: A mathematical model suggests collectivity and inconstancy enhance the efficiency of neuronal migration in the adult brain
Source: PLoS Comput Biol. 2025 Jun 5;21(6):e1013105. doi: 10.1371/journal.pcbi.1013105 (PMC12140228; doi:10.1371/journal.pcbi.1013105)
Supplement: S1 Appendix — (PDF) [file pcbi.1013105.s001.pdf]

S1 Appendix

I Biological experiments

Ia Mice

All animal experiments were performed in accordance with the guidelines and regulations of Doshisha University. The Dcx-DsRed mice were provided by Dr. Qiang Lu (Beckman Research Institute of the City of Hope) [1]. The Gfap-EGFP mice [2] were obtained from the Mutant Mouse Research Resource Center (MMRRC; Research Resource Identifier: MMRRC_000315-MU).

Ib Live cell imaging of in vitro cell culture

The V-SVZ was isolated from the Dcx-DsRed mice aged postnatal day 1–5 and cut into 100–200 µm-diameter pieces in cold Leibovitz's L-15 Medium (Wako, 128-06075). For live cell imaging of the collective migration of neuroblasts, the V-SVZ pieces were embedded into 70% Matrigel (Corning, 354234) diluted with Leibovitz's L-15 Medium and cultured on multiwell glass-bottom dishes (Matsunami, D141400) in Neurobasal medium (Thermo Fisher Scientific, 21103-049) containing 10% fetal bovine serum (Thermo Fisher Scientific, 10437-028), 2% MACS NeuroBrew-21 (Miltenyi Biotec, 130-097-263), 2 mM GlutaMAX Supplement (Thermo Fisher Scientific, 35050061), and 1% penicillin-streptomycin (Wako, 168-23191). The cultures were placed in a humidified 37°C, 5% CO_2 incubator and imaged one day later.

To track migration, the cells were treated with 10 µM of NucleoSeeing Live Nucleus Green (Funakoshi, FDV-0029) for 1 h, followed by medium replacement. Cells were imaged using a confocal microscope (Evident, FV3000) with a 20x objective lens (Evident, UPLXAPO 20X) every 4 min for 12 h in a stage-top incubator (BLAST) adjusted to 37°C, 5% CO_2. The nucleus of a cell was manually tracked using the MTrackJ plugin [3] in Fiji/ImageJ (NIH), and the moving velocity between frames was calculated.

For imaging a single migrating cell, the V-SVZ pieces were treated with 0.25% trypsin/EDTA (Wako, 201-16945) for 10 min at room temperature and then dissociated mechanically with a pipette in Dulbecco's Modified Eagle Medium (Wako, 044-29765) containing 10% fetal bovine serum and 40 µg/mL DNase (Sigma-Aldrich, 10104159001). The cell suspension was filtered through a 70 µm mesh cell strainer (Corning, 352350) and centrifuged at 1,000 $\times$ g for 10 min at 4°C for reaggregation. The cell aggregates were cultured as described above. Two days later, the cells were treated with 1 µg/mL Hoechst 33342 (Thermo Fisher Scientific, H1399) and imaged as described above.

Ic Imaging of neuroblasts in an injured brain

Using young adult male Gfap-EGFP mice aged 9–12 weeks, ischemic stroke was induced by the middle cerebral artery occlusion and brain sections were prepared at post-surgery day 18 as described previously [4]. The sections were incubated with guinea pig anti-Dcx (Sigma-Aldrich, AB2253) in blocking solution (10% normal donkey serum and 0.2% Triton X-100 in phosphate buffered saline) overnight at 4°C, followed by incubation with AlexaFluor 568 goat anti-guinea pig (Thermo Fisher Scientific, A-11075) in blocking solution for 2 h at room temperature. The sections were imaged using the FV3000 confocal microscope with the 20x objective lens.

II Mathematical modeling and simulations

We modeled neuroblasts and astrocytes in a two-dimensional field (see Sections 2.1 and 2.2 in the main text, respectively) with the contact-based interactions between them (Section IIa). Using the model, we performed the computer simulations (Section IIb) for neuroblasts (Section IIc) and astrocytes (Section IId).

IIa Modeling contact-based interactions between agents

Because an overview of contact-based interactions is explained with the summation terms in Section 2.3 (Eq 8), the details of each term are given below. A direct circle-to-circle interaction, the first term in Eq 8, is defined as follows:

$$\boldsymbol{f}_{kl}(t) = \{-k_{\mathrm{R}}(r_k(t) + r_l(t) - |\boldsymbol{x}_l(t) - \boldsymbol{x}_k(t)|) + H\}\boldsymbol{e}_{\mathrm{C},kl}(t), \tag{13}$$

where $\boldsymbol{e}_{\mathrm{C},kl}(t)$ represents the unit vector from $\boldsymbol{x}_k(t)$ to $\boldsymbol{x}_l(t)$. If the circle is an astrocytic unit, the radius of each circle ($r_k(t)$ and $r_l(t)$) follows Eq 7; if the circle is a soma or a tip, the radius is a positive constant, r_{S} or r_{T}, respectively. The positive constant k_{R} determines the strength of the repulsion and is multiplied by the overlapping length between circles k and l. The parameter $H \geq 0$ indicates the adhesion, which is a force opposite to repulsion (Fig 2c):

$$H = \begin{cases} H_{\mathrm{N}} & \text{between somas, tips, or a soma and a tip of different neuroblasts,} \\ H_{\mathrm{A}} & \text{between astrocytic units of different astrocytes,} \\ H_{\mathrm{a}} & \text{between astrocytic units in an astrocyte,} \\ 0 & \text{else,} \end{cases} \tag{14}$$

where H_{N} and H_{A} represent the strength of the chemical binding between neuroblasts and between astrocytes, respectively, and H_{a} represents the strength of the physical binding of a single astrocyte's units, which is substantially larger than H_{A}.

A via-process interaction—the second and third terms in Eq 8—is expressed by the following Eqs 3–5, which together illustrate that, for example, when an astrocytic unit contacts with a neuroblast's process at a position 30 µm away from the soma and 10 µm away from the tip, repulsion forces acting on the soma, tip, and astrocytic unit take a ratio of 10 : 30 : −40 (Fig 2d). When circle k (soma or tip) contacts circle l via circle k-linking process, the second term in Eq 8 acts on circle k as

$$\boldsymbol{g}_{kl}(t) = \{p(t)k_{\mathrm{R}}(r_l(t) + w - |\boldsymbol{x}_l(t) - \boldsymbol{x}_{\mathrm{P},kl}(t)|)\}\boldsymbol{e}_{\mathrm{P},lk}(t), \tag{15}$$

where the repulsion-related constant k_{R} is multiplied by a overlapping length between circle l and circle k-linking process; w is half the width of the process; $\boldsymbol{x}_{\mathrm{P},kl}(t)$ represents the coordinates of the perpendicular foot of $\boldsymbol{x}_l(t)$ on the midline of circle k-linking process; $\boldsymbol{e}_{\mathrm{P},lk}(t)$ is a unit vector from $\boldsymbol{x}_l(t)$ to $\boldsymbol{x}_{\mathrm{P},kl}(t)$ (Fig 2d). By $p(t)$, the repulsion strength is distributed according to the contact position of circle l onto the process:

$$p(t) = \begin{cases} \dfrac{|\boldsymbol{x}_{\mathrm{P},kl}(t) - \boldsymbol{x}_{k,\mathrm{T}}(t)|}{|\boldsymbol{x}_{k,\mathrm{S}}(t) - \boldsymbol{x}_{k,\mathrm{T}}(t)|} & \text{if circle } k \text{ is a soma,} \\ \dfrac{|\boldsymbol{x}_{\mathrm{P},kl}(t) - \boldsymbol{x}_{k,\mathrm{S}}(t)|}{|\boldsymbol{x}_{k,\mathrm{S}}(t) - \boldsymbol{x}_{k,\mathrm{T}}(t)|} & \text{if circle } k \text{ is a tip,} \end{cases} \tag{16}$$

which means that circle k is repelled more as circle l's contact with circle k-linking process approaches to circle k.

When circle k (soma, tip, or astrocytic unit) contacts circle l (soma or tip) via circle l-linking process, the third term in Eq 8 acts on circle k as follows:

$$\boldsymbol{g}'_{kl}(t) = \{-k_{\mathrm{R}}(r_k(t) + w - |\boldsymbol{x}_k(t) - \boldsymbol{x}_{\mathrm{P},lk}(t)|)\}\boldsymbol{e}_{\mathrm{P},kl}(t), \tag{17}$$

which is interpreted as a case of $p(t) = -1$ in Eq 3, with a difference of whether the process mediating between circles k and l is of circle k (Eq 3) or of circle l (Eq 5). Likewise, $\boldsymbol{x}_{\mathrm{P},lk}(t)$ represents the

coordinates of the perpendicular foot of $\boldsymbol{x}_k(t)$ on the midline of circle j-linking process, and $\boldsymbol{e}_{\mathrm{P},kl}(t)$ is a unit vector from $\boldsymbol{x}_k(t)$ to $\boldsymbol{x}_{\mathrm{P},lk}(t)$.

IIb General simulation condition

The model implemented in C++ (S1 File) was simulated using several different parameter sets (S1 Table) on a MacBook Pro Apple M3 Chip. Differential equations (Eqs 1,2,5,6) were solved using the Euler method. The time interval per step, dt, was set to 0.01 min with a maximum step of $T_{\max} = 900,000$, corresponding to $t_{\max} = 9,000$ [min] (150 h, 6.25 d). The modeled neuroblasts and astrocytes were distributed randomly in the simulation area with a square of 600×600 µm (Fig 3). A periodic boundary condition was applied to all of the four sides of the area. Accordingly, the coordinates of circle k (soma, tip, or astrocytic unit) were corrected as follows:

$$\boldsymbol{x}_k[x](t) = \begin{cases} \boldsymbol{x}_k[x](t) + 600 & \text{if } \boldsymbol{x}_k[x](t) < -300, \\ \boldsymbol{x}_k[x](t) - 600 & \text{if } \boldsymbol{x}_k[x](t) > 300, \end{cases} \quad \boldsymbol{x}_k[y](t) = \begin{cases} \boldsymbol{x}_k[y](t) + 600 & \text{if } \boldsymbol{x}_k[y](t) < -300, \\ \boldsymbol{x}_k[y](t) - 600 & \text{if } \boldsymbol{x}_k[y](t) > 300, \end{cases} \tag{18}$$

where $[x]$ and $[y]$ denote the x and y components of the two-dimensional vector, respectively. To calculate the positional relationship between two circles, the vector from circle k to circle l (soma, tip, or astrocytic unit), $\boldsymbol{v}_{kl}(t)$, was similarly corrected as

$$\boldsymbol{v}_{kl}[x](t) = \begin{cases} \boldsymbol{v}_{kl}[x](t) + 600 & \text{if } \boldsymbol{v}_{kl}[x](t) < -300, \\ \boldsymbol{v}_{kl}[x](t) - 600 & \text{if } \boldsymbol{v}_{kl}[x](t) > 300, \end{cases} \quad \boldsymbol{v}_{kl}[y](t) = \begin{cases} \boldsymbol{v}_{kl}[y](t) + 600 & \text{if } \boldsymbol{v}_{kl}[y](t) < -300, \\ \boldsymbol{v}_{kl}[y](t) - 600 & \text{if } \boldsymbol{v}_{kl}[y](t) > 300, \end{cases} \tag{19}$$

which involves the soma-tip interaction in a neuroblast, the distance to count neuroblasts near each astrocyte, and the contact-based interactions between circles.

To minimize the influence of initial randomness, ten trials were performed for each simulation condition, that is, $N_{\mathrm{trial}} = 10$. The m-th trial ($m = 1, 2, \ldots N_{\mathrm{trial}}$), in which the seed value for the pseudorandom number generator was set to $m - 1$, shared an initial state among the different simulation conditions. One trial with 40 astrocytes took approximately three minutes and required 200 MB memory.

IIc Parameters for neuroblasts

Let the number of neuroblasts $N_{\mathrm{N}} = 24$ (Fig 3a). The initial coordinates of the i-th neuroblast's soma, $\boldsymbol{x}_{i,\mathrm{S}}(0)$, were randomly generated. The tip was positioned near the soma of the same neuroblasts:

$$\begin{cases} \boldsymbol{x}_{i,\mathrm{T}}[x](0) = \boldsymbol{x}_{i,\mathrm{S}}[x](0) + d_{\mathrm{init}} \cos \theta_{\mathrm{init}}, \\ \boldsymbol{x}_{i,\mathrm{T}}[y](0) = \boldsymbol{x}_{i,\mathrm{S}}[y](0) + d_{\mathrm{init}} \sin \theta_{\mathrm{init}}, \end{cases} \tag{20}$$

where $[x]$ and $[y]$ denote the x and y components of the vector, respectively. The initial direction θ_{init} was randomly determined from the range $[0, 2\pi)$ rad. We let the soma-tip distance $d_{\mathrm{init}} = 1$ [µm]; the close positioning at the beginning prevented the phenomenon wherein several neuroblasts crossed the processes.

A previous in vitro study reported that the proportions of resting and moving neuroblasts at different times were very similar [5], implying that saltation cycles were not synchronized among cells. Accordingly, the phase of neuroblast i's process in the saltation cycle (Eq 3) shifted at regular intervals, that is, $\phi_{\mathrm{sal},i} = 2i\pi/N_{\mathrm{N}}$, $i = 1, 2, \ldots N_{\mathrm{N}}$. In the condition with a temporal change, the phase of neuroblast i's process in the activity cycle (Eq 9) was set identically, that is, $\phi_{\mathrm{act}} = 3\pi/2$.

The constant and inconstant cases were compared to test our hypothesis that the migration efficiency is improved by temporal change in the movement of neuroblasts as observed in vitro (Fig 4a). In the constant case, we set $A_{\mathrm{act}} = 0$ in Eq 9, such that the saltation amplitude of all neuroblasts constantly takes

$A_{\mathrm{sal}}(t) = A_{\mathrm{mid}} = 10$ [µm] (Fig 4b, black curve). As we set $L = 40$ [µm] in Eq 3, a constant neuroblast targets a length within the range of 30–50 µm in a cyclic manner. For periodic-inconstant saltation amplitude, we set $\omega_{\mathrm{act}} = \omega_{\mathrm{sal}}/72, \omega_{\mathrm{sal}}/36, \omega_{\mathrm{sal}}/18, \omega_{\mathrm{sal}}/9$ in Eq 9. If $A_{\mathrm{act}} = 5$ [µm] and $\omega_{\mathrm{act}} = \omega_{\mathrm{sal}}/9$, a neuroblast repeats nine saltation cycles per activity cycle while periodically changing its saltation amplitude within the range of 5–15 µm (Fig 4b, green curve). As we set $\omega_{\mathrm{sal}} = 2\pi/20$ [rad/min] in Eq 3, one saltation cycle requires 20 min; thus, one activity cycle in the case of $\omega_{\mathrm{act}} = \omega_{\mathrm{sal}}/9$ requires $20/60 \times 9 = 3$ [h]. In the case of random-inconstant saltation amplitude, we set $\sigma_{\mathrm{A}} = 1, 2, 3, 4, 5$ [µm] and $u = 1, 3, 9, 18, 36, 72$. The natural number u determines the number of saltation cycles that the neuroblast repeats at a random amplitude. We updated random numbers at the steps when the saltation phase $\omega_{\mathrm{sal}} t + \phi_{\mathrm{sal},i}$ (Eq 3) exceeded a multiple of $2\pi u$ to avoid sudden changes in the target length.

To explore another inconstant factor in the context of long-term activity, we changed the neuroblast adhesion strength H_{N} (Eq 14) periodically or randomly as implemented in $A_{\mathrm{sal}}(t)$ in Eqs 9 and 10. The periodic change in adhesion strength is expressed as

$$H_{\mathrm{N}}(t) = H_{\mathrm{mid}} + A_{\mathrm{H}} \sin(\omega_{\mathrm{act}} t + \phi_{\mathrm{act}}), \tag{21}$$

where H_{mid} and A_{H} are positive constants corresponding to the middle value and amplitude, respectively, in the long-term periodic change in the adhesion strength; ϕ_{act} is the initial phase of the common activity cycle. Under the condition with random adhesion strength, Eq 21 is altered to

$$H_{\mathrm{N}}(t) \sim \mathcal{N}(H_{\mathrm{mid}}, \sigma_{\mathrm{H}}), \tag{22}$$

where H_{mid} corresponds to the mean, and $\sigma_{\mathrm{H}} > 0$ is the standard deviation. The adhesion strength of all the neuroblasts is truncated within $[0, 2H_{\mathrm{mid}}]$ and changed at the steps when the phase $\omega_{\mathrm{sal}} t$ exceeds a multiple of $2\pi u$, where the natural number u determines the number of saltation cycles that the neuroblast repeats with a random value of the adhesion strength.

In the main simulations, we set a constant value for the adhesion strength between neuroblasts, that is, $H_{\mathrm{N}} = 2.22$ [nN]. For the supplementary simulations, we examined different constant values: $H_{\mathrm{N}} = 0, 0.56, 1.11, 1.67, 2.78, 3.33$ [nN]. Otherwise, we considered inconstant adhesion $H_{\mathrm{N}}(t)$ with middle strength $H_{\mathrm{mid}} = 2.22$ [nN]; $\omega_{\mathrm{act}} = \omega_{\mathrm{sal}}/36$ in the periodic case (Eq 21), and $u = 3$ in the random case (Eq 22). These supplementary trials were performed at a constant saltation amplitude $A_{\mathrm{sal}}(t) = A_{\mathrm{mid}} = 10$ [µm] (Eq 9).

IId Parameters for astrocytes

We set the number of astrocytes $N_{\mathrm{A}} = 0, 10, 20, 30, 40$ to test the effect of the astrocyte-dense activation state. The initial coordinates of unit 1 of astrocyte i, $\boldsymbol{x}_{i,1}(0)$, were randomly generated. The other two units were positioned next to the first unit of the same astrocyte to form an equilateral triangle:

$$\begin{cases} \boldsymbol{x}_{i,j}[x](0) = \boldsymbol{x}_{i,1}[x](0) + d_{\mathrm{init}} \cos \dfrac{2\pi(j-1)}{3}, \\ \boldsymbol{x}_{i,j}[y](0) = \boldsymbol{x}_{i,1}[y](0) + d_{\mathrm{init}} \sin \dfrac{2\pi(j-1)}{3}, \end{cases} \quad j = 2, 3, \tag{23}$$

where $[x]$ and $[y]$ denote the x and y components of the vector, respectively. We let the inter-unit distance $d_{\mathrm{init}} = 1$ [µm]; the close positioning at the beginning avoided the case in which a neuroblast's process was crossed with the linkage of a single astrocyte's units. The initial radius of astrocyte i's unit j was set to the minimum, that is, $r_{i,j}(0) = r_{\mathrm{min}}$.

We tested the significance of a biologically observed phenomenon in which astrocytes retract the processes in response to nearby neuroblasts [4,6]. This interaction was implemented in "reactive shrinkage" simulations, in which we let astrocytic units shrink depending on the number of nearby neuroblasts as shown in Eq 7 (Fig 3b). In contrast, in the "mismatched shrinkage" simulations, the target radius of an

astrocytic unit ($\bar{r}_{i,j}$) is determined by the number of nearby neuroblasts of another astrocytic unit ($B_{i',j'}$) so that apparently random astrocytic units shrink independently of the neuroblast positions (Fig 3c). For example, $\bar{r}_{1,1}$, $\bar{r}_{1,2}$, $\bar{r}_{1,3}$, and $\bar{r}_{2,1}$ are determined by $B_{N_{\mathrm{A}},3}$, $B_{N_{\mathrm{A}},2}$, $B_{N_{\mathrm{A}},1}$, and $B_{N_{\mathrm{A}}-1,3}$, respectively.

References

1. Wang X, Qiu R, Tsark W, Lu Q. Rapid promoter analysis in developing mouse brain and genetic labeling of young neurons by doublecortin-DsRed-express. J. Neurosci. Res., 2007;85(16):3567–3573. `https://doi.org/10.1002/jnr.21440`

2. Gong S, Zheng C, Doughty ML, Losos K, Didkovsky N, Schambra UB, et al. A gene expression atlas of the central nervous system based on bacterial artificial chromosomes. Nature, 2003;425(6961):917–925. `https://doi.org/10.1038/nature02033`

3. Meijering E, Dzyubachyk O, Smal I. Methods for cell and particle tracking. Methods Enzymol., 2012;504:183–200. `https://doi.org/10.1016/B978-0-12-391857-4.00009-4`

4. Kaneko N, Herranz-Pérez V, Otsuka T, Sano H, Ohno N, Omata T, et al. New neurons use Slit-Robo signaling to migrate through the glial meshwork and approach a lesion for functional regeneration. Sci. Adv., 2018;4(12):eaav0618. `https://doi.org/10.1126/sciadv.aav0618`

5. Wichterle H, García-Verdugo JM, Alvarez-Buylla A. Direct evidence for homotypic, glia-independent neuronal migration. Neuron, 1997;18(5):779–791. `https://doi.org/10.1016/s0896-6273(00)80317-7`

6. Kaneko N, Marín O, Koike M, Hirota Y, Uchiyama Y, Wu JY, et al. New neurons clear the path of astrocytic processes for their rapid migration in the adult brain. Neuron, 2010;67(2):213–223. `https://doi.org/10.1016/j.neuron.2010.06.018`
