# Supplementary material for: A mathematical model suggests collectivity and inconstancy enhance the efficiency of neuronal migration in the adult brain
Source: PLoS Comput Biol. 2025 Jun 5;21(6):e1013105. doi: 10.1371/journal.pcbi.1013105 (PMC12140228; doi:10.1371/journal.pcbi.1013105)
Supplement: S1 Table — (PDF) [file pcbi.1013105.s002.pdf]

S1 Table. Simulation parameters.

Parameter	Specification	Unit	Value	Equation(s)
k_{P}	spring constant of a neuroblast's process	[N/m]	* 1.67 $\times 10^{-4}$ (S1 Fig)	(1,2)
L	middle length of a neuroblast's process	[m]	4.00×10^{-5}	(3)
ω_{sal}	angular velocity of a process in neuroblast saltation	[rad/s]	$1.67\pi \times 10^{-3}$	(3)
μ_{low}	smaller friction coefficient of a neuroblast's soma and tip	[Ns/m]	3.33×10^{-2}	(4)
μ_{high}	larger friction coefficient of a neuroblast's soma and tip	[Ns/m]	3.33×10^{-1}	(4)
μ_{A}	friction coefficient of an astrocytic unit	[Ns/m]	* 1.67 (S1 Fig)	(5)
τ	time constant determining how slow an astrocytic unit's radius reaches the target radius	[s]	* 2.00×10^{3} (S1 Fig)	(6)
r_{max}	maximum radius of an astrocytic unit	[m]	* 3.30×10^{-5} (S2 Fig)	(7)
ρ_{A}	coefficient for an astrocytic unit's target radius determined by its nearby neuroblast number	[m]	* 3.30×10^{-6} (S1 Fig, S2 Fig)	(7)
r_{min}	minimum radius of an astrocytic unit	[m]	* 1.65×10^{-5} (S2 Fig)	(7)
d_{slit}	reacheable distance of the Slit signal of a neuroblast	[m]	* 1.50×10^{-5} (S1 Fig)	(7) related
A_{mid}	middle (mean) amplitude of a process in neuroblast saltation	[m]	1.00×10^{-5}	(9)
A_{act}	amplitude of a process in neuroblast activity in the periodic-inconstant condition of saltation amplitude	[m]	* 0 or 5.00×10^{-6} (Fig 7, S4 Fig)	(9)
ω_{act}	angular velocity of a process in neuroblast activity in the periodic-inconstant condition	[rad/s]	* $4.63\pi \times 10^{-5}$ (S4 Fig)	(9,21)
ϕ_{act}	initial phase of a process in neuroblast activity in the periodic-inconstant condition of saltation amplitude	[rad]	1.50π	(9,21)
σ_{A}	standard deviation of saltation amplitude under a normal distribution in the random-inconstant condition	[m]	* 5.00×10^{-6} (S4 Fig)	(10)
u	number of saltation cycles by which saltation amplitude values are updated in the random-inconstant condition	—	* 3 (S4 Fig)	(10,22) related
k_{R}	coefficient for repulsion strength determined by two agents' overlapping length	[N/m]	8.33×10^{-3}	(13,15,17)
r_{S}	radius of a neuroblast's soma	[m]	* 5.00×10^{-6} (S2 Fig)	(13,15,17) related
r_{T}	radius of a neuroblast's tip	[m]	* 3.00×10^{-6} (S2 Fig)	(13,15,17) related
H_{N}	adhesion strength between different neuroblasts	[N]	* 2.22×10^{-9} (Fig 9)	(14,21,22)
H_{A}	adhesion strength between different astrocytes	[N]	* 5.56×10^{-9} (S1 Fig)	(14)
H_{a}	adhesion strength between astrocytic units in a single astrocyte	[N]	1.67×10^{-7}	(14)
w	half the width of a neuroblast's process	[m]	* 2.00×10^{-6} (S2 Fig)	(15,17)
H_{mid}	middle (mean) adhesion strength between two somas, two tips, or a soma and a tip when inconstant	[N]	2.22×10^{-9}	(21,22)
A_{H}	amplitude in neuroblast activity in the periodic-inconstant condition of neuroblast adhesion strength	[N]	* 0 or 2.22×10^{-9} (S5 Fig)	(21)
σ_{H}	standard deviation of neuroblast adhesion strength under a normal distribution in the random-inconstant condition	[N]	1.11×10^{-9}	(22)

* Changed depending on simulation conditions. The value used in the main simulations is shown. See the parenthesized figure for the variation.
