# Supplementary figures and images for: A mathematical model suggests collectivity and inconstancy enhance the efficiency of neuronal migration in the adult brain
Source: PLoS Comput Biol. 2025 Jun 5;21(6):e1013105. doi: 10.1371/journal.pcbi.1013105 (PMC12140228; doi:10.1371/journal.pcbi.1013105)

# S1 Fig

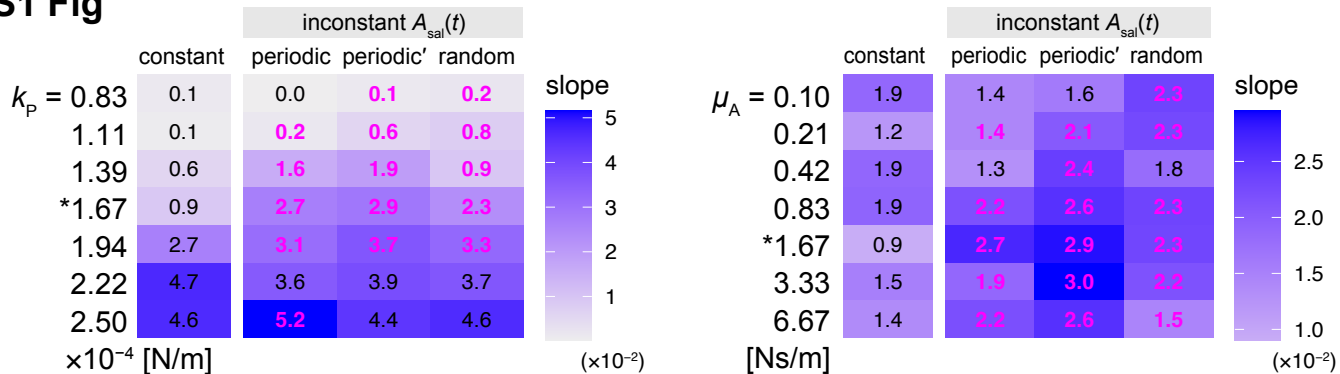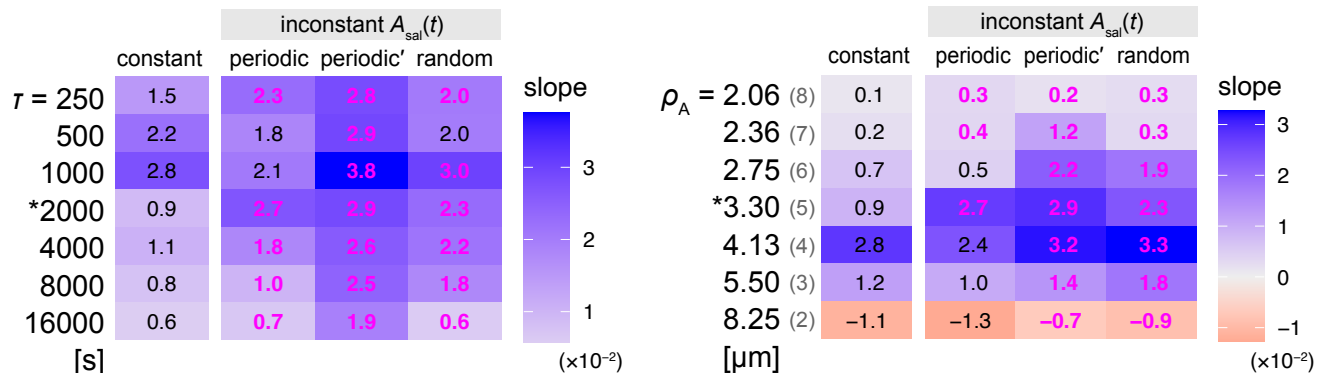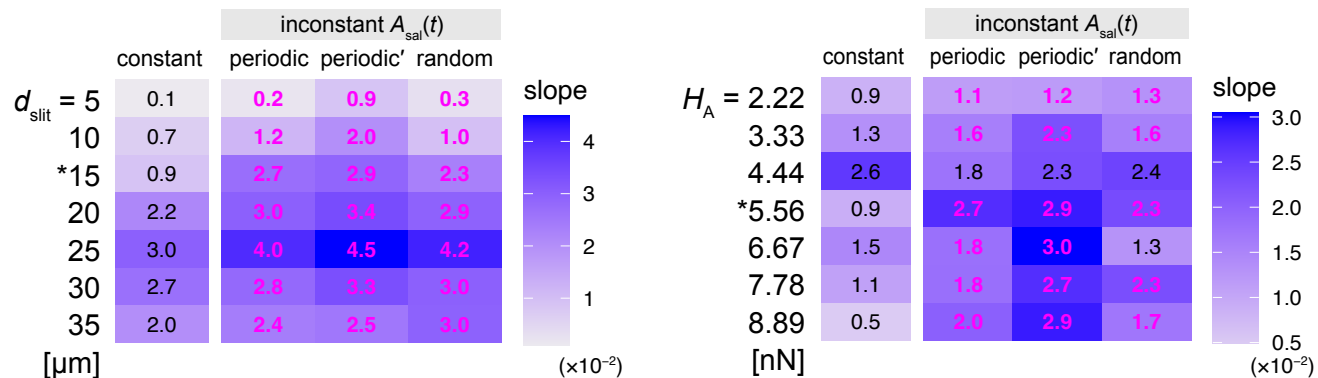

Supplement: S1 Fig — The neuroblast-related parameter kP (top left) and the astrocyte-related parameters μA (top right), τ (middle left), ρA (middle right), dslit (bottom left), and HA (bottom left) are tested. The values of each parameter are shown on the left side of the heat map, and the asterisk indicates the value used in the main simulations and when the other parameters were altered. The parenthesized numbers for ρA represent the lowest number of neuroblasts that causes an astrocytic unit to target the minimum radius. The “constant” column indicates the conditions where neuroblasts have a constant adhesion strength, that is, Aact=0. The “periodic” and “periodic ′ ” columns indicate periodic-inconstant conditions with Aact=5 and Aact=10, respectively, both with ωsal/ωact=36. The “random” column indicates random-inconstant conditions with σA=5 and u = 3. The number of astrocytes showing reactive shrinkage is NA=40. A large positive value on a blue background indicates that a neuroblast in a collective tends to be faster than a solitary neuroblast; a large negative value on a red background indicates the opposite tendency. The bold magenta value in the inconstant condition indicates a slope value greater than that under the constant condition for a common value of each tested parameter, representing the collective-advantageous tendency of inconstancy. (PDF) [file pcbi.1013105.s003.pdf]

## S2 Fig

### Neuroblasts without swelling

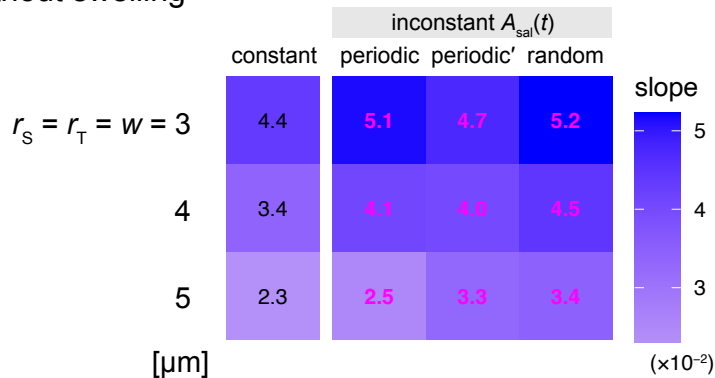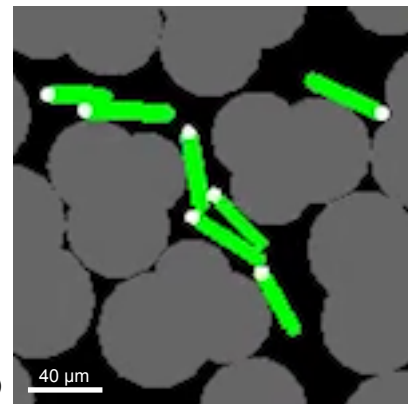

### One astrocytic unit per astrocyte

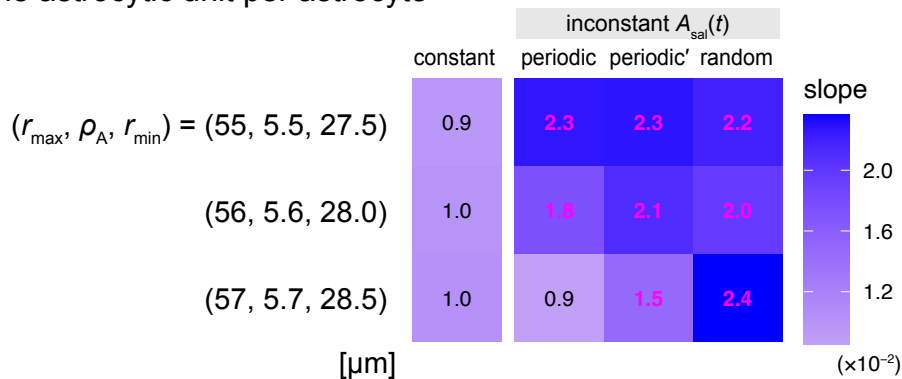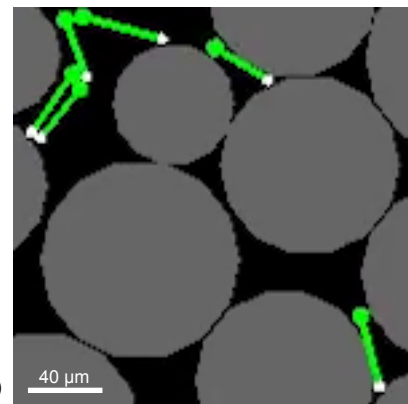

Supplement: S2 Fig — Top: rounded-rectangular neuroblasts with the same values of rS, rT, and w (5 μm in the snapshot). Bottom: exact-circle astrocytes, each with one astrocytic unit (rmax=56, ρA=5.6, and rmin=28 in the snapshot). Size-related values are listed on the left side of each heat map. Heat maps are shown as in S1 Fig. The number of astrocytes showing reactive shrinkage is NA=40. (PDF) [file pcbi.1013105.s004.pdf]

**S3 Fig**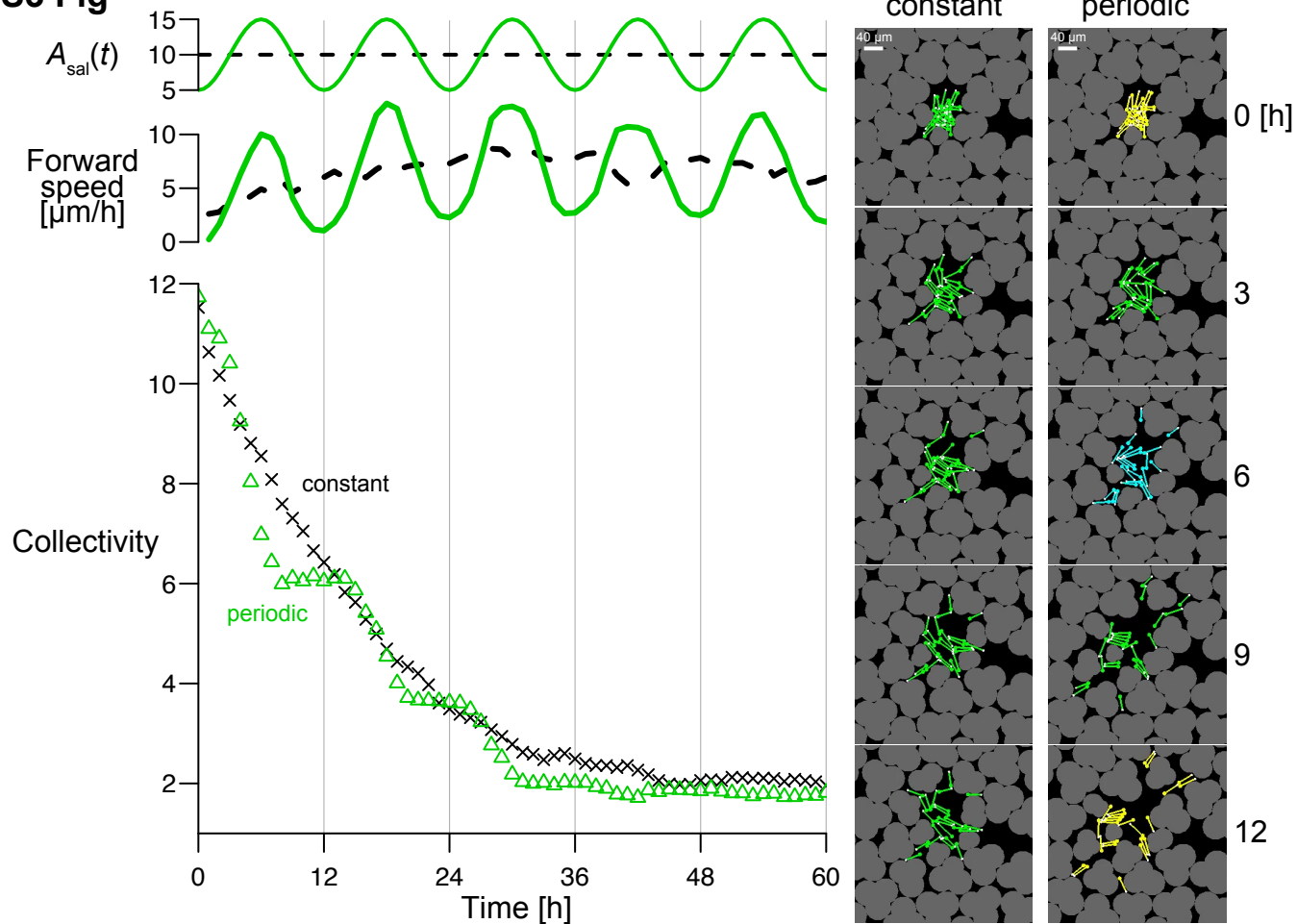

Supplement: S3 Fig — The forward speed vi(t) and collectivity ni(t) over time are averaged over ten trials. Target length Asal(t) and forward speed: dashed lines, constant; solid lines, periodic-inconstant. Collectivity: cross, constant; triangle: periodic-inconstant. In the inconstant condition, the wavelength of the activity cycle is set to 12 h, that is, Aact=5 and ωsal/ωact=36. The yellow, green, and cyan colors of the neuroblasts in the snapshots indicate the minimum, average, and maximum states in Asal(t), respectively. The number of astrocytes showing reactive shrinkage is NA=40. (PDF) [file pcbi.1013105.s005.pdf]

# S4 Fig

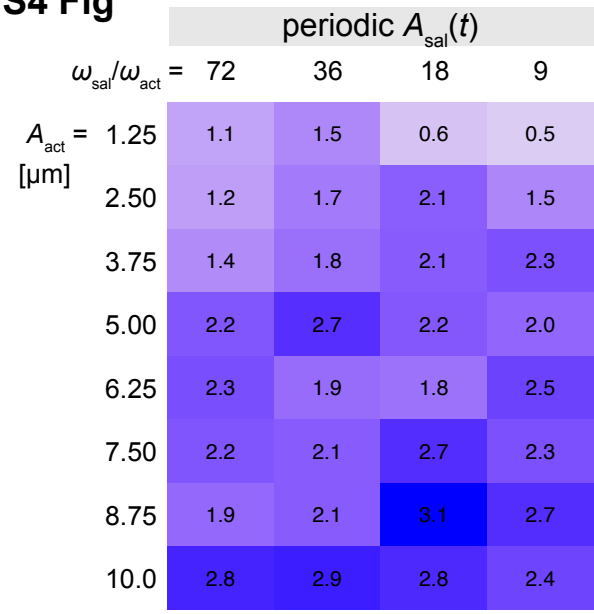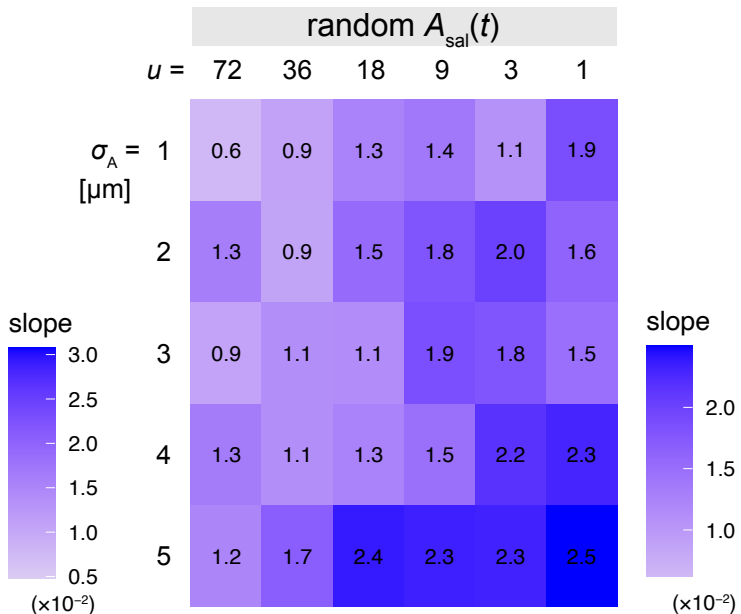

Supplement: S4 Fig — Left: the values at the top indicate the number of saltation cycles per activity cycle (ωsal/ωact), whereas those on the left indicate the activity amplitude (Aact). Right: values at the top indicate the number of saltation cycles that the neuroblast repeats with a value of saltation amplitude (u), whereas those on the left indicate the standard deviation of the random change in saltation amplitude (σA). The number of astrocytes showing reactive shrinkage is NA=40. The adhesion strength between neuroblasts is constant (HN=2.22 [nN]). A large positive value on a blue background indicates that a neuroblast in a collective tends to be faster than a solitary one. (PDF) [file pcbi.1013105.s006.pdf]

# S5 Fig

inconstant  $H_N(t)$

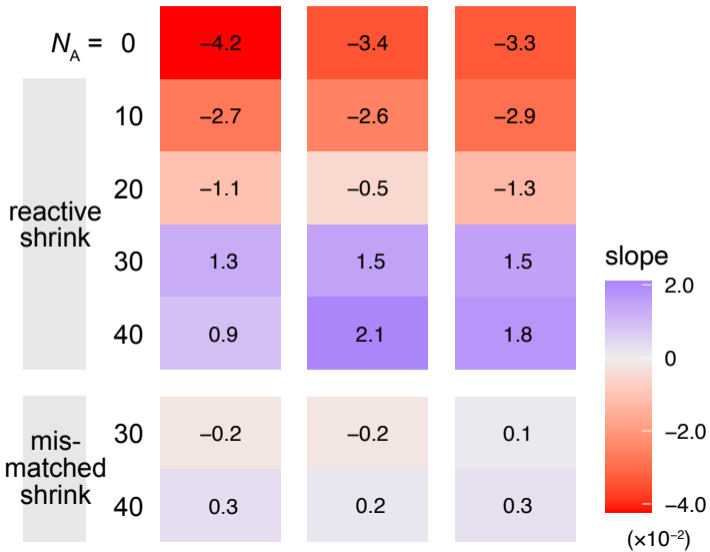

Supplement: S5 Fig — Values on the left indicate the number of astrocytes. Upper rows labeled “reactive shrink” show conditions in which astrocytic units shrink depending on the number of nearby neuroblasts. Lower rows labeled “mismatched shrink” show the conditions where apparently random astrocytic units shrink. The left column indicates the conditions where neuroblasts have a constant adhesion strength, that is, AH=0. The middle column indicates periodic-inconstant conditions with AH=8 and ωsal/ωact=36. The right column indicates random-inconstant conditions with σH=4 and u = 3. The saltation amplitude is constant in these conditions (Asal(t)=Amid=10). A large positive value on a blue background indicates that a neuroblast in a collective tends to be faster than a solitary one, while a large negative value on a red background represents the opposite tendency. (PDF) [file pcbi.1013105.s007.pdf]
