# Supplementary material for: A mathematical model suggests collectivity and inconstancy enhance the efficiency of neuronal migration in the adult brain
Source: PLoS Comput Biol. 2025 Jun 5;21(6):e1013105. doi: 10.1371/journal.pcbi.1013105 (PMC12140228; doi:10.1371/journal.pcbi.1013105)
Supplement: Attachment — Submitted filename: Response.pdf [file pcbi.1013105.s014.pdf]

Dear Editorial Board of PLOS Computational Biology,

Thank you very much for taking your time to handle our manuscript entitled "A mathematical model suggests collectivity and inconstancy enhance the efficiency of neuronal migration in the adult brain." We thoroughly revised the text, figures, and supplementary information according to the reviewers' valuable suggestions.

Reviewer #1:

#1-general) This manuscript entitled "A mathematical model suggests collectivity and inconstancy enhance the efficiency of neuronal migration in the adult brain" investigates computationally collective behaviors of migrating neuroblasts in a crowd of astrocyte, after proposing the simplified mathematical model of this neuroblast-astrocyte system. Their computational simulations firstly demonstrates that their model reproduces neuroblast collective behaviors with which they can migrate faster than solitary neuroblasts in the densely populated astrocytes, and after that, investigated several features of this collective behavior. Although the model proposed in this manuscript is simple or the computations may not be technically heavy, I still would like to value the significance of this study. Firstly, I acknowledge that the first major result of this manuscript -- the computational demonstration of the power of collectiveness of neuroblasts (or the motile elements) to efficiently move through the crowds of astrocytes (or obstacles) (Sect. 3.1) -- provides the solid progress in this field. While I think this result is intuitively reasonable, it does not mean it is trivial. Secondly, as one of the results around the collective neuroblast migration, the prediction of the importance of the inconstant neuroblast behavior for the effective collective migration (Sect. 3.3) is interesting. Thus, this manuscript could be a good candidate for publication. That being said, I would like to reserve the recommendation until the authors address the following points (at least, the major comments):

- RESPONSE: We thank reviewer #1 for the time to review our manuscript and the encouraging comments. We would like to respond point by point to the reviewer's suggestions below. The page and line numbers in this letter follow the manuscript that highlights our changes from the original submission (not the clean manuscript).

Major comments.

#1-major1) 1. In their model, the cells are made of the connected circles. In this case, because the peripheries of the elements have convex and concave shape, the two cells contacting each other may have the friction in between them when they slide relatively. I think this is not the effect which

the authors desired in the model and not something controllable. I cannot judge if this effect brings something significant in their results. So, I wonder if the authors could the authors evaluate if this effect is negligible to their results and comment it in the manuscript, or if they could improve the model to avoid such bumpy periphery.

- RESPONSE: This is an interesting perspective. For a modeled neuroblast, we performed new simulations where each neuroblast was smoothened into a rounded rectangle by setting the diameters of the soma and tip identically to the width of the process ($r_S = r_T = w$). As shown in new S2 Fig, the advantage of collectivity and inconstancy was consistent. We have kept the bumpy morphology in the main simulations because we referred to the actual morphology with a swelling soma and a busy tip and suppose that these convex or intricate parts would cause a friction when they slide in vivo. For a modeled astrocyte, we have confirmed that the tendency was reproduced even when an astrocyte is modeled as an exact circle (i.e., one astrocytic unit per cell; new S2 Fig). In this case, because each astrocyte shrinks concentrically, a single neuroblast often benefits from a cluster of neuroblasts on the other side of the astrocyte. Considering it unnatural, we have kept the main simulations with three astrocytic units per astrocyte to express the partial shrinkage. We have added the result statements related to smoothening detailly in Section 3.1 as below:

  > "Moreover, the advantage remained consistent when each neuroblast was smoothened into a rounded rectangle ($r_S = r_T = w$) and when each astrocyte was simplified into an exact circle with a single astrocytic units (S2 Fig), indicating that the frictional effect of the agents' concave and convex profiles was negligible on the results." (Results, Section 3.1, page 17, lines 349-353)

#1-major2) 2. Most of the mathematical explanations of their model is currently put only in the supplementary file, not in the main text (Section 2). However, actually, this is making the manuscript difficult to follow smoothly. Considering this is the journal for "computational" biology, the authors may try to reorganize the manuscript and move the essential equations into the main text. For example, at least, I would put the equations of motions of the cells [Eqs. (7), (8) and (11) in the supplementary text], time-dependent friction to make each neuroblast motile [Eq. (9) in the supplementary text] and the decomposition form of the cell-cell interaction [Eq. (12) in the supplementary text] into the main text. (Regarding Eq. (12) in the supplementary text, this decomposition form of the interaction itself, F= sum f_kl + sum g_kl + sum g'_kl, may be moved to the main text, but the definition of each interaction term, f_kl, g_kl, g'_kl, would be explained by only the words in the main text whereas the detailed math would be still kept in the supplementary text.)

This is because, without having these equations, the readers who have mathematical/physical backgrounds may feel difficulties to even just imagine what kind of principles were used to model their dynamics.

- RESPONSE: We agree with this suggestion. We have thoroughly revised Sections 2.1-2.3 while moving several equations from S1 Appendix to the main text: the neuroblast-related equations (new Eqs 1,2,4), the astrocyte-related equations (new Eqs 5,6), and the decomposition form of interaction (new Eq 8). Along with this, we moved all the neuroblast/astrocyte modeling to the main text, while keeping the detailed definition of each interaction term in S1 Appendix as the reviewer suggested.

#1-major3) 3. The authors may expand Section 3.3 to discuss what is the cause of the efficient collective behavior by the inconstant saltatory amplitude. Actually, they discuss it a bit in the Discussion (Section 4.2) by the sentence "[…] transient large-amplitude movements contributed to deadlock breaking, which increased the efficiency of the forward movement of the large neuroblast clusters (Fig. 6d)", but this is just by words and Fig. 6d is just a snapshot. The authors may need to provide more quantifications or new simulations to support this proposition.

- RESPONSE: We agree with this suggestion. Firstly, we have added videos corresponding to the snapshots in Figs 6 and 8 (new S2 and S4 Videos, respectively) since only showing snapshots was not very informative as the reviewer mentioned. Secondly, we have performed new simulations in which all the neuroblasts were initially clustered at the center of the simulation field. S3 Fig shows that neuroblasts reduced collectivity when the saltation amplitude was transiently large. We have written a relevant sentence in Section 3.3 as follows:

> "In additional simulations in which all of the neuroblasts were initially clustered at the center of the field, the neuroblast cluster reduced collectivity when the saltation amplitude was large (S3 Fig)." (Results, Section 3.3, page 18, lines 375-378)

Minor comments.

#1-minor1) Page 5 line 79 --- I guess that phi_i may be a typo, and it actually should be phi_{sal,i}

- RESPONSE: We have corrected it (page 7, line 132). We sincerely appreciate the careful review.

#1-minor2) Page 8 line 141-152 --- I could not get the logic flow in between these two sentences. Maybe the authors forgot to put the sentence like “Each of these aspects (i)-(iv) can be explained in more details as follows.” or the phrase/sentence like that? I might be wrongly understanding what they intended.

- RESPONSE: As the reviewer suggested, we have added a sentence in advance of the individual explanations: “Each of these aspects i)--iv) is explained in detail as follows” (page 12, line 239).

#1-minor3) Page 8 line 158-163 “By contrast, […] located (Fig. 3c)” --- I could not understand these sentences. There might be some typos?

- RESPONSE: We agree that the explanation appeared to be misunderstanding and complicated. We have refined the relevant explanation as follows: “In contrast, in the mismatched shrinkage condition, when a neuroblast approaches a certain astrocytic unit, a randomly chosen unit instead of the nearby unit shrinks (Fig 3c).” (page 12, lines 256-259)

#1-minor4) Abstract, Introduction, etc. --- Apparently, to introduce a motivation of this computational study, the authors are frequently emphasizing that the observations of this system are challenges so the mathematical simulation study is a good alternative. However, I do not feel this is efficient reasoning of this study because the mathematical model used in this manuscript is pretty simplified one (2D only, cells as connected circles, etc) and there are no guarantees whether this model can quantitatively reproduce the experimental observations. And, if they really would like to emphasize so, they may need more rigorous comparisons of their model and experimental quantifications in this manuscript. Please note that, nevertheless, I do not want to undermine the significance of the study by this mathematical model. I think this model study is indeed providing the interesting concept, as mentioned above. So, I rather would like to suggest the authors to update the introduction so that it does not sound as if they want to study this mathematical model as an alternative of actual experiments but to predict something new which we had not realized.

- RESPONSE: We agree with this important viewpoint. In the last paragraph of Introduction, we have included the suggested viewpoint and also emphasized the advantage of simplified models as below:

> “Mathematical models are not merely an alternative to biological systems, but are superior in predicting new ideas, which might be biologically validated in the future. One major prediction from our simulations was} that inconstancy (temporal change) in

the movement of neuroblasts improves migration efficiency, which has received little attention in biological studies of cell migration." (Introduction, pages 5-6, lines 91-95)

"Upon modeling, we placed importance on simplicity, which allows us to easily extract a key mechanism for the aspect in focus, in contrast to a complicated system in which numerous factors simultaneously affect each other." (Introduction, page 5, lines 85-87)

Reviewer #2:

#2-general) This paper presents a mathematical model simulating the behavior of neuroblast migration in the adult brain, focusing on their collective behavior and interaction with astrocytes. The authors developed a two-dimensional model incorporating saltatory movement of neuroblasts, astrocyte shrinkage responses, and cell-cell interactions. Their simulations suggest that collective migration is advantageous in dense astrocyte environments, and temporal variations in neuroblast behavior ("inconstancy") can enhance migration efficiency.

Strengths:
1. Novel contribution to understanding collective cell migration mechanisms, particularly highlighting the role of "inconstancy" using mathematical simulation
2. Well-structured model incorporating key biological features (saltatory movement, Slit-Robo signaling, cell adhesion)
3. Potential applications in both medical research and swarm robotics

Weakness:
1. Two-dimensional model may not fully capture the complexity of three-dimensional brain tissue
2. There is very limited discussion on model sensitivity.
3. The astrocyte response is very simplified. The author only used a linear response to approximate the astrocyte dynamics and only consider the shrinkage behavior. However, actual astrocytes have highly complex, star-shaped morphology, exhibiting highly dynamic process extensions and retractions. Multiple signaling pathways may be involved.
4. There is a overemphasis on mathematical parameters without broader discussion of potential biological variations in neuroblast populations and various biological processes.

- RESPONSE: We thank reviewer #2 for taking the time to review our manuscript. The summary of the strengths and weakness is very helpful to us. The weakness points 1 and 3 would together

raise a concern that our model is too simple to capture the complex neuroblast-astrocyte system. However, simplicity is a major priority in our modeling. One advantage of a simplified model is that we can easily extract a key mechanism for one aspect that we would like to focus. For example, the Kuramoto model is a simplified mathematical model that has been extended to explain many phenomena in the aspect of synchronization among oscillators, represented by the cases of metronomes and fireflies (e.g., Lizarraga & de Aguiar, Chaos, 2020). In the manuscript, we have revised the last paragraph of Introduction to emphasize the advantage of simplified models as below:

> "Upon modeling, we placed importance on simplicity, which allows us to easily extract a key mechanism for the aspect in focus, in contrast to a complicated system in which numerous factors simultaneously affect each other." (Introduction, page 5, lines 85-87)

The page and line numbers in this letter follow the manuscript that highlights our changes from the original submission (not the clean manuscript). We would like to respond to the other weakness points in the followings.

Major concerns:

#2-major1) 1. The model's reliance on mathematical parameters, which are hand-tuned rather than experimentally derived, limits its biological applicability. Additionally, the findings may not readily translate to human neurobiology, where neuroblast migration is limited compared to rodents. To address the concern, the author should declare how reasonable the parameter setting is and add additional sensitivity analysis.

- RESPONSE: We agree that the reasonability of the parameter setting was poorly written. We referred to biological observations to determine the parameters related to cell morphology. However, we have a difficulty in deriving many of the other parameters from biological experiments with the present techniques. For example, we have not developed the technology to accurately measure the spring constant of the neuroblast process and the magnitude of force on cellular adhesion. This is why we have hand-tuned such parameters in silico while checking if visualized simulations agreed with biological observations. In the manuscript, we have additionally mentioned the rationale of some parameter values and our considerations for hand-tuning as follows:

> "We used biological knowledge [8, 17, 22] and our observation (Fig 1) to determine parameters for neuroblast morphology such as the average process length L = 40 [µm],

the average saltation amplitude Amid = 10 [μm], the radii of the soma and tip (r_S = 5 [μm] and r_T = 3 [μm], respectively), and half the width of the process (w = 2 [μm]) (S1 Table). Based on the kinematics (Fig 1a), we set the wavelength of the saltation cycles to 20 min, that is, $\omega_{sal} = 2\pi/20$ [rad/min]. We determined the astrocyte size such that the width of the astrocyte territory was approximately 100 μm, tuning the maximum radius of astrocytic units r_{max} and the adhesion strength between astrocytic units (H_a). We manually tuned the other parameters because of the technical difficulty in quantitative measurements, as represented by the spring constant of the neuroblast process k_P and the intercellular adhesion strength (H_N between neuroblasts and H_A between astrocytes). Upon manual tuning, we confirmed whether the visualized simulations agreed with the biological observations. For example, neuroblasts hardly form chains with a large value of k_P or small value of H_N, which disagrees with in vivo situations. To reinforce the reliance, we investigated the robustness of the results using a range of parameter values." (Methods, Section 2.4, page 11, lines 218-233)

We also agree with the necessity of some sensitivity analysis and thus performed additional simulations with a range of parameter variations. We have confirmed that our results (e.g., advantages of collectivity and inconstancy) remained mostly consistent in various conditions, as newly written in Sections 3.1 and 3.3 with the new supplementary figures (S1 and S2 Figs):

"The collectives were robustly advantageous in a range of parameter variations, such as the spring constant of a neuroblast process k_P, the time constant for astrocyte shrinkage τ , the extent of astrocyte shrinkage determined by the number of nearby neuroblasts ρ_A, the distance of the slit signal d_{slit}, and the adhesion strength between astrocytes H_A (S1 Fig, positive slope values in the 'constant' columns). Moreover, the advantage remained consistent when each neuroblast was smoothened into a rounded rectangle ($r_S = r_T = w$) and when each astrocyte was simplified into an exact circle with a single astrocytic unit (S2 Fig), thus indicating that the frictional effect of the agents' concave and convex profiles was negligible on the results." (Results, Section 3.1, page 17, lines 345-353)

"A range of parameter values mostly resulted in the advantage of collectives with an inconstant saltation amplitude compared to those with a constant amplitude (S1 Fig, S2 Fig), indicating the robustness of the results." (Results, Section 3.3, page 19, lines 389-392)

As the reviewer pointed out, neuroblast migration is more limited in the human brain than

in rodents. Therefore, developing strategies to enhance neuroblast migration will be crucial for effective neuronal regeneration in humans. While previous studies have primarily focused on factors that promote individual cell migration, the biological significance of collective migration remains largely unexplored due to the technical challenges of analyzing complex cell behaviors in collectives. Given that V-SVZ-derived neuroblasts migrate collectively to navigate the environment with the densely packed neuronal fibers and glia cells, our study provides insights that could inform strategies to enhance neuronal migration in humans. To address this, we have added an explanation of collective neuroblast migration in Introduction and discussed the potential clinical applications of our findings in Perspectives, as follows:

> "While the RMS begins to develop before birth, the distinct "chain migration" pattern takes approximately three weeks to become fully established [13]. In the adult brain, V-SVZ-derived neuroblasts, the only cell type capable of long-distance migration, also exhibit chain migration as they travel toward lesion sites [8]. Although the biological significance of this migration pattern is not fully understood, it has been suggested that collective chain migration provides neuroblasts with an advantage in navigating the challenging and less accessible adult brain environment, characterized by densely packed neuronal fibers and glial cells." (Introduction, page 3, lines 20-27)

> "Although neuroblast migration in the adult brain has been observed in various animal species, including some primates, evidence of neuroblast migration in the adult human brain remains limited [41, 42]. However, postmortem brain studies have reported enhanced cell proliferation in the V-SVZ and the presence of newly generated neurons near lesion sites [43, 44]. Enhancing the migration of endogenous or transplanted neural progenitor cells toward injured sites promoted functional recovery in mouse model for ischemic stroke and traumatic brain injury [8, 19, 20, 45]. Additionally, neural progenitor cell transplantation improved functional outcomes in Huntington's disease [46, 47] and cognitive function in mouse models for Alzheimer's disease models [48, 49]." (Discussion, Section 4.4, page 25, lines 549-557)

> "For clinical applications, neuronal regeneration in the large human brain requires newly generated neurons, whether endogenous or transplanted, in order to effectively migrate from the origin to cover the injured areas. The microenvironment varies depending on the disease and its progression. Reactive astrocytes, as modeled in this study, proliferate in conditions such as ischemic stroke, neurodegenerative diseases, and traumatic brain injury; however, tissue changes following injury differ according to disease type, stage,

and severity [50, 51]. By incorporating these environmental factors, our model could predict neuronal migration dynamics across various pathological conditions, thus offering insights into the distribution and settlement of transplanted cells. This may aid in optimizing transplantation sites and timing, as well as in selecting extracellular interventions to improve therapeutic outcomes." (Discussion, Section 4.4, page 25, lines 558-568)

#2-major2) 2. The simulated results are intriguing but fall short of being compelling or inspiring from a biological perspective without stronger connections to experimental evidence. There is insufficient discussion of how the simulated outcomes align with or explain in vivo and in vitro observations. This disconnect weakens the biological relevance and interpretability of the study, the authors should detail in the results section how the simulated findings correspond to specific experimental data. For instance, they could compare the model's predictions with observed patterns in neuroblast migration under similar conditions in existing studies or suggest how their predictions might guide future experiments.

➢ RESPONSE: We acknowledge that the original manuscript did not sufficiently describe the connection between our mathematical model and existing biological studies. While this paper includes limited biological data, numerous studies have detailed the behavior of V-SVZ-derived neuroblasts migrating in the adult brain. We have contributed to this research field through several publications (Kaneko et al., Neuron, 2010; Fujioka et al., EBioMedicine, 2017; Minegishi et al., Cell Rep., 2018; Kaneko et al., Sci. Adv., 2018; Ohno et al., Biomaterials, 2023; Nakajima et al., Nat. Neurosci., 2024). Due to the limitations of current experimental technique, directly measuring and controlling physical parameters in collectively migrating neuroblasts remains challenging. Thus, in our mathematical model, parameters were adjusted to replicate key features of their unique collective migration patterns. In response to the review's suggestion, we have revised Introduction to better characterize neuroblast migration and have refined Discussion to more explicitly relate our simulation results to biological observations, as follows:

> "Within these chain-like collectives, neuroblasts form discontinuous adherens junctions with one another, as revealed by electron microscopy [6]. Intercellular adhesion within these chains is regulated by various molecules including N-cadherin and PSA-NCAM [14, 15]. Live imaging of migrating neuroblasts in acute brain slices and extracellular matrix-enriched gels has demonstrated that the cells within a chain frequently exchange neighbors during migration [16, 17], thus indicating that intercellular adhesion is dynamically remodeled." (Introduction, page 4, lines 39-45)

“Both periodic and random changes in neuroblast properties increased the forward movement of neuroblasts in large collectives, but not of solitary migrating neuroblasts (Figs 7a,d, 9a, S4 Fig, S5 Fig). Physiologically, the V-SVZ-derived neuroblast migration in postnatal and adult brains is controlled by multiple hormones, neurotransmitters, and neurotrophic factors [28–30]. In general, these factors are transiently released in response to stimulations that include physiological events, such as food intake and sleep, and to neuronal/physical activation. For example, neuroblast migration is promoted by ghrelin, a hormone secreted by the intestine in response to food intake [29]. The neurotransmitter serotonin released from activated serotonergic neurons promotes neuroblast migration in the RMS and the path toward lesions in the neocortex [28]. Interestingly, serotonin secretion is periodically altered by circadian rhythms. Therefore, several extrinsic factors affect the inconstant migration behavior of neuroblasts over multiple timescales in vivo, which in turn may contribute to the efficient migration of neuroblasts in adult brain tissue.” (Discussion, Section 4.3, pages 23-24, lines 507-520)

#2-major3) 3. While the paper considers a range of factors (e.g., adhesion strength, astrocyte density, inconstancy in neuroblast behavior), it is unclear if these are comprehensive or represent the full biological complexity influencing neuroblast migration. The omission of potentially significant biological interactions or environmental conditions raises concerns about the realism and applicability of the simulated results to actual biological systems. To address this, the authors should either a) substantiate that the selected factors are indeed the primary determinants of neuroblast migration or b) explicitly discuss the potential limitations of the factors considered and how unaccounted variables might influence the outcomes.

- RESPONSE: In this study, we focused on several factors that have been shown to play critical roles in regulating neuroblast migration. Numerous biological studies indicate that adhesion between neuroblasts, as well as their interactions with surrounding cells, is tightly regulated to enable efficient collective migration. Based on this evidence, we selected intercellular adhesion strength and astrocyte density (major glial cell type in injured brain tissue) as key parameters in our mathematical model. However, to maintain a manageable level of complexity in our simulations, we excluded certain known factors involved in neuroblast migration, such as blood vessels as migration scaffolds and secreted factors influencing migration direction. In response to the reviewer’s suggestion, we explicitly discussed the potential limitations of our model in Discussion:

"In the brain, migrating neuroblasts are guided by various diffusible and non-diffusible factors supplied by surrounding cells and circulating fluids (e.g., blood and cerebrospinal fluid). In contrast, because our model did not impose any directional control on the neuroblast movement (see Section 4.1), the alignment between colliding neuroblasts in the simulations emerged passively through physical interactions between their elongated bodies." (Discussion, Section 4.1, page 21, lines 431-436)

"Our mathematical model does not incorporate mechanisms for directional control in order to allow for a range of migration scenarios. This simplification is a limitation of this study. In other models of collective cell migration, such as the lateral line of zebrafish embryos, melanoma cells in mice, and neural crest cells in frog embryos, collective formation makes each cell faster and more directed, which may involve a chemotactic gradient formed by the cells at the rear of the collective [40]. Neuroblasts generated in the adult V-SVZ often form elongated clusters, occasionally along blood vessels, during long-range migration toward the injury site [4, 22, 24, 25]. Incorporating such rear-to-front signaling mechanisms and scaffold interactions into future models may be able to provide deeper insights into the collective migration process in neuronal regeneration." (Discussion, Section 4.4, pages 24-25, lines 535-548)

Minor concerns:

#2-minor1) 1. It may be better if there are more than 10 repetitions.

➢ RESPONSE: We have confirmed that the tendencies of the main plots (Figs 6b-d and 8b) are consistent even when the number of trials (repetitions) is reduced from 10 to 5, as shown in the lower figure (colors/symbols as in the original panels). We thus suppose that the sample size of 10 trials is reliable enough and performing more than ten trials would yield similar results.

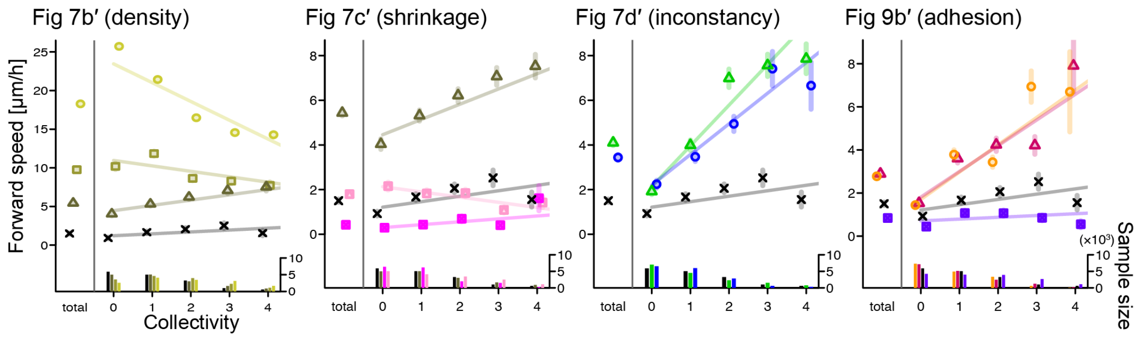

#2-minor2a) 2. Some technical points are unclear, for example, a) Missing details on numerical

integration methods and time-step selection

- RESPONSE: There have been the statements in the beginning of Section IIb (S1 Appendix): “The differential equations (Eqs 1,2,5,6) were solved using the Euler method. The time interval per step, dt, was set to 0.01 min.”

#2-minor2b) b) Unclear handling of periodic boundary conditions,

- RESPONSE: We have added the explanation in Section IIb of S1 Appendix.

#2-minor2c) c)No specification of computational requirements (runtime, memory usage)

- RESPONSE: We have added the statements in Section IIb (S1 Appendix): “on a MacBook Pro Apple M3 Chip” at the beginning and “One trial with 40 astrocytes took approximately three minutes and required 200 MB memory” at the last.

#2-minor3a) 3. Some figure presentiation could be refined: for example, a)Inconsistent scale bars and time stamps (especially in Figs 1, 6, and 8),

- RESPONSE: In Figs 1, 6, and 8, the scale for the snapshots within each figure has been consistent. To avoid misunderstanding, we have copied the scale bar to each panel in Figs 6 and 8 (and 3 too). For time stamps, we have refined Figure 1 to have consistent intervals. As for the simulations in Figs 6 and 8, however, we have kept the original time stamps because the key events to be emphasized often occur irregularly. For example, in Fig 6a, the long interval 0-48 h is to emphasize the static situation of the isolated neuroblast, whereas the short interval 48-51 h is to display the transient chain formation by newly coming neuroblasts. We hope that annotated intervals beside the arrows help the readers understand. Instead, we have added videos that correspond to the snapshots in Figs 6 and 8 to clarify the time scale.

#2-minor3b) b) Missing statistical significance indicators in regression analyses (Fig 7)

- RESPONSE: We agree with the importance of such indicators. However, unlike biological experiments, we can perform a limitless number of simulations. When we could obtain a large sample size as much as we want, any difference (no matter how slight) could turn to a statistically significant difference. For example, under a random number generator following a normal distribution with standard deviation 1, student's t test mostly returns no significant difference

between 100 samples with mean 0 and those with mean 0.001 but often returns a significant difference between 10,000,000 samples with mean 0 and those with mean 0.001. We thus refrain from indicating statistical significance for the simulation results.

#2-minor4) 4. More discussion could be made on implications for neurodegenerative disease therapies and linking predictions to existing biological knowledge.

- RESPONSE: According to the reviewer's suggestion, we have expanded the Perspectives section to discuss the therapeutic implications of our findings for neurodegenerative diseases and ischemic stroke (page 25, lines 549-568). Neural stem cell transplantation has been shown to improve functional recovery in mouse models of Huntington's disease (Reidling et al., Stem Cell Reports, 2018; Holley et al., Mol. Ther., 2023) and enhance cognitive function in Alzheimer's disease (Tong et al., J. Neurosci., 2014; Strell et al., Cell Transplant., 2023). For clinical applications, successful regeneration in the larger human brain requires newly generated neurons to migrate efficiently from their site of production or transplantation to effectively cover the injured area. Regulating the collective behavior of migrating neuroblasts, as demonstrated in our study, may extend migration distances and improve the efficacy of cell-based therapies. We hope the revised manuscript addresses your concerns.

Reviewer #3:

General comments:

#3-general) In this manuscript, Wakita et al. presents a computational framework to understand how the neuronal migration process in the adult brain. They explore and propose a possible model by which neuroblasts achieve efficient migration through collective behavior and temporal variability. Using in silico modeling and experimental observations, the study highlights key mechanisms underlying neuroblast migration, including the role of astrocytic interaction, neuroblast collectivity, and behavioral inconsistency. The text and figures are relatively easy and clear to follow. However, while the idea is interesting, I did not see biological interpretation of the results discussed or implications of the work inferred; a major concern is how effectively a mathematical model can simulate neuronal migration from a biological perspective.

- RESPONSE: We thank reviewer #3 for the time to review our manuscript. The reviewer's query is indeed important. We would like to respond point by point to the issues. The page and line numbers in this letter follow the manuscript that highlights our changes from the original

submission (not the clean manuscript).

Major issues:

#3-major1) 1. Reliability of the Mathematical Model: The foundation of any mathematical model predicting biological effects depends significantly on the robustness and reliability of the simulation. This, in turn, relies heavily on the integration of a substantial amount of biological data (e.g., images from this manuscript). However, the biological data presented here seems quite limited. Would it be possible to utilize published datasets from the literature as input to test the fidelity of this model? Additionally, could this model be tested using data on radial migration during embryonic development before applying it to the adult brain? Although the authors have emphasized the model's application to the adult brain, the extensive data available on embryonic development could serve as a valuable standard for testing.

- RESPONSE: We appreciate the reviewer's insightful comments regarding the reliability of our mathematical model. We recognize that the biological data included in this paper are limited. However, extensive studies have documented the migration of V-SVZ-derived neuroblasts in the adult brain, and we have investigating their migration behaviors in detail in our previous work (Kaneko et al., Neuron, 2010; Fujioka et al., EBioMedicine, 2017; Minegishi et al., Cell Rep., 2018; Kaneko et al., Sci. Adv., 2018; Ohno et al., Biomaterials, 2023; Nakajima et al., Nat. Neurosci., 2024).

  Following the reviewer's suggestion, we have reinforced the robustness and reliability of the results by performing new simulations with a wide range of parameter values, which mostly reproduced the principal tendencies for the advantage of collectivity and inconstancy (new S1 and S2 Figs; page 17, lines 345-353; page 19, lines 389-392). Although we have tried to tune several morphology-related parameters based on the biological knowledge, there are limited biological datasets for other characteristics. For example, we have a limitation to accurately measure the spring constant of the neuroblast process and the magnitude of force on cellular adhesion with the present techniques. For this reason, we have hand-tuned many parameters in silico while checking if visualized simulations agreed with biological observations. We have additionally explained such background in page 11, lines 218-233 (Section 2.4). We hope that a novel prediction from our simulations such as the advantage of inconstancy will be biologically validated in future, as newly written in Introduction (page 5, lines 91-93).

  Regarding the reviewer's suggestion to test the model using radial migration data from embryonic development, "radial migration" is indeed one of the most extensively studied

neuronal migration patterns in the brain. In the developing neocortex, neurons typically migrate individually, likely due to the relatively sparse cellular environment looser tissue connectivity. However, our study focuses on the significance of collective neuroblast migration in the densely packed mature brain, where neuronal fibers and glial cells provide a highly structured environment. Since our primary goal to develop strategies for promoting regeneration in the adult brain, we prioritize modeling neuroblast migration under these conditions. To clarify this focus, we have added the following sentences:

> "During migration, each neuroblast exhibits saltatory movement, characterized by the extension of a long leading process in the direction of travel, followed by somal translocation (Fig 1a, S1 Video) [4]. Neuronal migration has been extensively studied in the context of neocortical morphogenesis, in which excitatory and inhibitory neuron precursors migrate individually from the germinal zones at the ventricular surface toward the developing neocortex via radial and tangential migrations, respectively [10]. In contrast, neuroblasts in adult brains migrate collectively, maintaining close contact and forming elongated clusters referred to as "chains" [6, 11, 12] (Fig 1b). While the RMS begins to develop before birth, the distinct "chain migration" pattern takes approximately three weeks to become fully established [13]. In the adult brain, V-SVZ-derived neuroblasts, the only cell type capable of long-distance migration, also exhibit chain migration as they travel toward lesion sites [8]. Although the biological significance of this migration pattern is not fully understood, it has been suggested that collective chain migration provides neuroblasts with an advantage in navigating the challenging and less accessible adult brain environment, characterized by densely packed neuronal fibers and glial cells." (Introduction, page 3, lines 12-27)

> "Given that the promotion of neuroblast migration enhances neurological recovery in mice after brain injury [8, 19, 20], understanding the underlying mechanism and biological significance of migration mode tuning of neuroblasts will help develop a new strategy for successful neuronal regeneration. However, this issue remains largely unexplored because of the technical challenges in consistently observing individual neuroblasts within a chain over an extended period and in manipulating neuroblast motility and environmental factors with precision." (Introduction, page 5, lines 65-71)

#3-major2) 2. in vivo vs. in vitro Data: The manuscript primarily relies on in vitro culture and brain slice experiments as input for computational simulations. While the authors highlight the similarities between in vivo and in vitro systems, these approaches are less representative of the in

vivo environment, particularly for classical neuronal migration pathways, such as radial migration (cortical development) and tangential migration (adult neurogenesis), which are traditionally studied in vivo. Could publicly available in vivo datasets be incorporated to train the mathematical models? This would allow for further validation using the in vitro data, rather than the reverse approach.

- RESPONSE: We recognize the potential gap between in vivo and in vitro data in this research field, and we agree that referencing previously published in vivo datasets could be valuable. Following the reviewer's suggestion, we have explored public datasets available for training of our model. However, due to technical limitations, high-quality datasets capturing collective migration of V-SVZ-derived neuroblasts in vivo through live imaging have not yet published. This challenge arises from the anatomical location of the physiological migration route, the RMS, which is situated deep within the brain (very far from the cortical surface), making it difficult to visualize using light microscopy. Additionally, imaging neuroblasts in fibrous or edematous tissues following injury is further complicated by optical constraint. Instead, both our group and others have previously demonstrated the collective migration of adult-born neuroblasts in the RMS (tangential migration) and toward injury sites in vivo by labeling the newborn cells with thymidine analogues or virus vectors (Kirschenbaum et al., J. Neurosci., 1999; Grade et al., PLoS One, 2013; Kaneko et al., Sci. Adv., 2018). These studies have quantitatively analyzed their distribution, morphology and molecular mechanisms using both light and electron microscopy. We have carefully compared these in vivo data with findings from acute brain slices and cultured neuroblasts to ensure consistency and biological relevance (Minegishi et al., Cell Rep., 2018; Nakajima et al., Nat. Neurosci., 2024; Yamashita et al., J. Neurosci., 2006).

  In this study, we developed a simplified mathematical model to investigate the significance of collective neuroblast migration, carefully adjusting parameters to reflect key features of migration observed in these previous in vivo studies. Furthermore, regarding the interaction between migrating neuroblasts and surrounding astrocytes in our mathematical model, we have previously demonstrated this phenomenon not only with in vitro and ex vivo brain slice data but also with substantial in vivo evidences (Kaneko et al., Neuron, 2010; Fujioka & Kaneko et al., EBiomedicine, 2017; Kaneko et al., Sci. Adv., 2018).

  That said, we understand the reviewer's concern regarding the lack of direct in vivo dataset integration in our model. In the revised manuscript, we have mentioned previous in vivo studies about neuroblast migration in the adult brain more precisely, and added citations of those previous works in Introduction. We hope these additions clarify our approach and adequately address the reviewer's concerns.

#3-major3) 3. Incorporating Biological Factors into the Model: The observations regarding neuroblast migration—such as the cellular and molecular factors involved—are indeed interesting. How do the authors plan to incorporate these biological factors comprehensively into the proposed mathematical model? Once established, could this model be used to predict biological phenotypes and provide valuable guidance for mechanistic studies in disease contexts?

- RESPONSE: We appreciate the reviewer's interest in our simulation of neuroblast migration. As suggested, we aim to utilize this model to advance cell-based neuronal regeneration therapies in the future, although further studies are needed.

Transplantation of neural stem cells into the striatum has been shown to enhance functional recovery in mouse models of ischemic stroke and Huntington's disease (Yuan et al., Stem Cell Res. Ther., 2013; Kaneko et al., Sci. Adv., 2018; Reidling et al., Stem Cell Reports, 2018). Additionally, cell transplantation is increasingly being explored as a potential therapeutic strategy for other neurodegenerative diseases and traumatic brain injuries (Tong et al., J. Neurosci.; 2014; Strell et al., Cell Transplant. 2023; Zhu et al., Nat. Commun., 2019). Given the invasive nature of transplantation procedures, the number of cells and sites available for transplantation is inherently limited. Therefore, ensuring efficient migration of transplanted neurons from the injection site across the affected brain region is critical for therapeutic success. Importantly, the microenvironment varies significantly depending on the pathological condition and its progression. While reactive astrogliosis, modeled in this study, is commonly observed in conditions such as ischemic stroke, neurodegenerative diseases, and traumatic brain injury, its extent and distribution vary widely across different pathologies and individuals. Other tissue changes, including edema, fibrosis, and necrosis, also exhibited substantial variability. By incorporating these environmental factors, our model may help predict neuronal migration patterns under different disease conditions and stages. For example, it could estimate how effectively transplanted cells disperse from the injection site and where they are likely to settle, providing valuable insights for optimize transplantation strategies. Additionally, the model could guide the selection of extracellular interventions, such as administration of cell-attractive or trophic factors and the use of biomaterial scaffolds, to optimize distribution of neuroblasts and improve therapeutic outcomes. In response to the reviewer's comment, we have added a discussion of these potential applications to the Perspectives section, as follows:

"Enhancing the migration of endogenous or transplanted neural progenitor cells to

injured sites promoted functional recovery in mouse models of ischemic stroke and traumatic brain injury [8, 19, 20, 45]. In addition, neural progenitor cell transplantation improved functional outcomes in Huntington's disease [46, 47] and cognitive function in mouse models of Alzheimer's disease models [48, 49]. For clinical applications, neuronal regeneration in the large human brain requires newly generated neurons, whether endogenous or transplanted, in order to effectively migrate from the origin to cover the injured areas. The microenvironment varies depending on the disease and its progression. Reactive astrocytes, as modeled in this study, proliferate in conditions such as ischemic stroke, neurodegenerative diseases, and traumatic brain injury; however, tissue changes following injury differ according to disease type, stage, and severity [50, 51]. By incorporating these environmental factors, our model could predict neuronal migration dynamics across various pathological conditions, thus offering insights into the distribution and settlement of transplanted cells. This may aid in optimizing transplantation sites and timing, as well as in selecting extracellular interventions to improve therapeutic outcomes." (Discussion, Section 4.4, page 25, lines 553-568)

Minor Comments:

#3-minor) 1. Typographical Error in S1 Appendix Ic: There appears to be an inconsistency in the experimental description. The text states, "Using old male Gfap-EGFP mice aged 9–12 weeks, ischemic stroke was induced by the middle cerebral artery occlusion and brain sections were prepared at postnatal day 18 as described previously." This seems contradictory, as it would not be possible to prepare brain sections at postnatal day 18 in mice aged 9–12 weeks. This may be a copy-paste error. The description should likely read: "Using old male Gfap-EGFP mice aged 9–12 weeks, ischemic stroke was induced by middle cerebral artery occlusion, and brain sections were prepared at post-surgery day 18 as described previously."

- RESPONSE: We thank the reviewer for pointing out the typo in our paper. We have corrected the sentence in S1 Appendix Ic. We sincerely appreciate the reviewer's careful review and valuable contribution.

We sincerely appreciate the editor's and reviewers' time and thoughtful feedback, which have helped us improve our manuscript.

Sincerely yours,
Daiki Wakita, Yuriko Sobu, Naoko Kaneko, and Takeshi Kano

Correspondence:

Takeshi Kano, M.D., Ph.D.
Professor
School of Systems Information Science,
Future University Hakodate
116-2 Kamedanakano-cho, Hakodate, Hokkaido, Japan 041-8655
Tel: +81-138-34-6425
E-mail: tkano@fun.ac.jp

Co-correspondence:

Naoko Kaneko, M.D., Ph.D.
Professor
Graduate School of Brain Science,
Doshisha University
1-3 Tatara Miyakodani, Kyotanabe, Kyoto, Japan 610-0394
E-mail: nkaneko@mail.doshisha.ac.jp
