# Supplementary material for: A mathematical model suggests collectivity and inconstancy enhance the efficiency of neuronal migration in the adult brain
Source: PLoS Comput Biol. 2025 Jun 5;21(6):e1013105. doi: 10.1371/journal.pcbi.1013105 (PMC12140228; doi:10.1371/journal.pcbi.1013105)
Supplement: Attachment — Submitted filename: Response_auresp_2.pdf [file pcbi.1013105.s015.pdf]

Dear Editorial Board of PLOS Computational Biology,

Thank you very much for taking your time to handle our manuscript entitled "A mathematical model suggests collectivity and inconstancy enhance the efficiency of neuronal migration in the adult brain." We revised the manuscript according to the reviewers' suggestions.

Reviewer's Responses to Questions

#1) Reviewer #1: In the revised version, the authors addressed all my comments in detail. I found the manuscript has been very much improved.

- RESPONSE: We thank reviewer #1 for the time to review our manuscript again. We are honored to have incorporated the reviewer's valuable opinions into our manuscript.

#2-general) Reviewer #2: Overall, the authors have substantially improved the manuscript by addressing many of my concerns, particularly regarding parameter sensitivity analysis, model robustness, and potential clinical applications. The addition of supplementary simulations and videos enhances the presentation of their findings. Most of my previous concerns have been carefully addressed. I only have several extra comments that the author may take to make the manuscript more readable:

- RESPONSE: We thank reviewer #2 for the time to review our manuscript again. We would like to respond point by point to the reviewer's suggestions below. The page and line numbers in this letter follow the manuscript that highlights our changes from the original submission (not the clean manuscript).

#2-1) 1. For the previous major concern 1, while the authors acknowledge hand-tuning parameters, they provide insufficient justification for why their selected parameter values lead to optimal biological behavior. I would recommend adding quantitative criteria for parameter selection and comparative evidence showing why these specific values best reproduce biological observations. Or, if the quantitative criteria experiment is difficult to perform, a more systematically description should be made in the main text on how the author actually "check visualized simulations agreed with biological observations" and why so.

- RESPONSE: We agree that the description for manual tuning was poor. Because of biological limitations on quantitative criteria experiments, we have followed the second suggestion. We thus have newly inserted Section 2.4 (Parameter setting) before Section 2.5 (Simulation conditions) and described the way of manual tuning in more detail in the second paragraph:

"We manually tuned the other parameters because of the technical difficulty in quantitative measurements. Upon manual tuning, we confirmed whether the visualized simulations agreed with the biological observations. To enable neuroblasts to alternately anchor the soma and tip for forward movement, smaller and larger friction coefficients (μ_{low} and μ_{high}) need to be sufficiently small and large, respectively. When neuroblasts collide with other neuroblasts, they hardly form a chain-like collective under a large value of the spring constant of the process (k_P) or small value of the intercellular adhesion strength between neuroblasts (H_N); in the opposite case, neuroblasts form a huge cluster incapable of displacement. We thus chose the intermediate values for these parameters to allow for chain formation with aspect ratios similar to those in vitro, i.e., the long axis three to four times longer than the short axis [22]. To keep astrocytes stationary even when pushed by neuroblasts, k_P and the friction coefficient of astrocytic units (μ_A) were set sufficiently small and large, respectively. The intercellular adhesion strength between astrocytes (H_A) needs to be small enough for neuroblasts to pass between the astrocytes, but not zero given the biological basis [26]. The parameters related to the extent and rate of astrocyte shrinkage (τ, ρ_A, r_{min}, and d_{slit}) were determined based on in vitro videos showing neuroblast-astrocyte interactions [8]. For example, astrocytes appear to retract their processes when neuroblasts are touching or close enough to touch, but not when neuroblasts are far away; we thus let the reachable distance of the Slit signal reasonably short (d_{slit} = 15 [μm]). The coefficient for repulsion strength (k_R) was set large enough so that agents do not overlap or cross each other, but small enough so that astrocytic units within an astrocyte do not split. Because the manually tuned values are not biologically definitive, we investigated the robustness of the results using a range of parameter values to reinforce the reliance." (2 Methods, 2.4 Parameter setting, pages 10-11, lines 206-229)

#2-2) 2. In terms of writing, I think the author may consider adding some of the response to my previous major concern 3 (about why the certain important factors are selected) to the main text, before the introduction of mathematical formulation,

- RESPONSE: This is a great idea to inform the readers of the general focus of our model in advance. Based on our previous response to reviewer #2's comment (#2-major3 in the last letter), we have revised the beginning of the Methods section as below.

"We focused on several factors that have been shown to play critical roles in regulating neuroblast migration, such as adhesion between neuroblasts as well as their interactions with the surrounding cells. Based on these findings, we selected, for example, intercellular adhesion strength and astrocyte density as key parameters. We implemented

these parameters into a mathematical model in which the neuroblast-astrocyte system was simplified on a two-dimensional field. In our assumption, neuroblasts are migratory agents (Section 2.1); astrocytes are environmental agents (Section 2.2); they make contact-based interactions (Section 2.3)." (2 Methods, page 5, lines 70-77)

#2-3) 3. Just curious, is there any other molecular signals beyond Slit-Robo might be involved in the process? If there is, shouldn't they be included in the discussion part?

➢ RESPONSE: We thank the reviewer for the thoughtful comment and interest in the potential expandability of our neuroblast-astrocyte interaction model. In addition to Slit-Robo signaling, several other pathways—such as laminin-β1 integrin and Eph-ephrin signaling—have been reported to be involved in interactions between neuroblasts and astrocytes in modulating astrocyte morphology. Following the reviewer's suggestion, we have addressed this point in the revised manuscript (Section 4.4) as follows:

> "Additionally, beyond Slit-Robo signaling, other molecular mechanisms, such as laminin-β1 integrin signaling and various Eph-ephrin signaling pathways, have been reported to mediate interactions between neuroblasts and the surrounding astrocytes [11,41]. Incorporating these signaling pathways may further enhance our understanding of the mechanisms underlying neuroblast migration and neuronal regeneration." (4.4 Perspectives, page 24, lines 537-542)

#3) Reviewer #3: The authors' revisions have addressed my concerns.

➢ RESPONSE: We thank reviewer #3 for the time to review our manuscript again. We are honored to have incorporated the reviewer's valuable opinions into our manuscript.

Journal Requirements:

Please confirm whether your study includes live participants. If so, please insert an Ethics Statement at the beginning of your Methods section, under a subheading 'Ethics Statement'. It must include:
i) The full name(s) of the Institutional Review Board(s) or Ethics Committee(s)
ii) The approval number(s), or a statement that approval was granted by the named board(s).

➢ RESPONSE: We have inserted Section 2.7 (Ethics statement) at the end of the Methods section. If we insert the statement at the beginning of the Methods section, it disconnects the logical flow from the general concept of our mathematical model (the paragraph immediately after the Methods header) to the subsections for detailed modeling. Thus, to maintain readability, we have

written the ethics statement not at the beginning but at the end of the Methods section. We would appreciate your understanding in this matter.

We sincerely appreciate the editor's and reviewers' time and thoughtful feedback, which have helped us improve our manuscript.

Sincerely yours,
Daiki Wakita, Yuriko Sobu, Naoko Kaneko, and Takeshi Kano

Correspondence:

Takeshi Kano, M.D., Ph.D.
Professor
School of Systems Information Science,
Future University Hakodate
116-2 Kamedanakano-cho, Hakodate, Hokkaido, Japan 041-8655
Tel: +81-138-34-6425
E-mail: tkano@fun.ac.jp

FUTURE UNIVERSITY HAKODATE

Co-correspondence:

Naoko Kaneko, M.D., Ph.D.
Professor
Graduate School of Brain Science,
Doshisha University
1-3 Tatara Miyakodani, Kyotanabe, Kyoto, Japan 610-0394
E-mail: nkaneko@mail.doshisha.ac.jp

Doshisha University
Graduate School of Brain Science
